# A model to study complement involvement in experimental retinal degeneration

**DOI:** 10.1080/03009734.2018.1431744

**Published:** 2018-02-13

**Authors:** Camilla Mohlin, Kerstin Sandholm, Anders Kvanta, Kristina N. Ekdahl, Kjell Johansson

**Affiliations:** a Linnaeus University Faculty of Health and Life Science, Linnaeus Center of Biomaterials Chemistry, Linnaeus University, Kalmar, Sweden; b Department of Clinical Neuroscience, Section for Ophthalmology and Vision, St. Erik Eye Hospital, Karolinska Institutet, Stockholm, Sweden; c Department of Immunology, Genetics and Pathology, Rudbeck Laboratory, Uppsala, Sweden; d School of Medical Sciences, Örebro University, Örebro, Sweden

**Keywords:** AMD, complement system, ocular diseases, retina, RPE

## Abstract

**Background:**

The complement system (CS) plays a role in the pathogenesis of a number of ocular diseases, including diabetic retinopathy (DR), glaucoma, uveitis, and age-related macular degeneration (AMD). Given that many of the complex eye-related degenerative diseases have limited treatment opportunities, we aimed to mimic the *in vivo* retinal degenerative process by developing a relevant co-culture system.

**Method and materials:**

The adult porcine retina was co-cultured with the spontaneously arising human retinal pigment epithelial cells-19 (ARPE-19).

**Results:**

Inflammatory activity was found after culture and included migrating microglial cells, gliosis, cell death, and CS activation (demonstrated by a minor increase in the secreted anaphylotoxin C3a in co-culture). CS components, including C1q, C3, C4, soluble C5b-9, and the C5a receptor, were expressed in the retina and/or ARPE cells after culture. C1q, C3, and CS regulators such as C4 binding protein (C4BP), factor H (CFH), and factor I (CFI) were secreted after culture.

**Discussion:**

Thus, our research indicates that this co-culturing system may be useful for investigations of the CS and its involvement in experimental neurodegenerative diseases.

## Introduction

The complement system (CS), an essential element of innate immunity, is an organized complex network of more than 50 soluble and surface proteins, primarily produced in the liver, that are activated through proteolytic cascades (1,2). The main purpose of the CS is to recognize foreign pathogens and eliminate misfolded molecules and dying cells (3). The CS is divided into at least three independent, interactive activation pathways: the classical pathway (CP), the lectin pathway (LP), and the alternative pathway (AP). In the absence of stimuli, complement proteins circulate in an inactive form; when stimulated, the appropriate proteases cleave, activate, and amplify the complement cascade. CS activation leads to opsonization of foreign surfaces, the generation of anaphylatoxins such as C3a and C5a, which are active in the immune response (2), and may end with targeted lysis by the membrane attack complex (MAC) (1,2). The AP is in a continuous active low-level state under physiological conditions and needs to be tightly controlled by endogenous regulators to avoid tissue damage (4). Both soluble and membrane-bound regulators are needed to control the CS at various levels.

The CS has been shown to contribute to a diverse set of ocular diseases, including diabetic retinopathy (DR), glaucoma, autoimmune uveitis (AU), and age-related macular degeneration (AMD). These diseases, significant contributors to visual impairment and blindness worldwide, have been shown to be attenuated by complement inhibition *in vivo* (5,6). In DR, retinal neurons and vessels become compromised, and an early para-inflammation in combination with a sustained hyperglycemic environment can induce CS activation (7,8) in an effort to retain homeostasis. Diabetes dysregulates the CS (8,9) and may affect the production as well as the function of membrane-bound complement regulators such as CD59 (10). Autoantibodies against endogenous oxidized, glycated, mislocated proteins can activate the CP (8,11), and extracellular advanced glycation end-products (AGEs) can function as neo-epitopes for mannose-binding lectin (MBL) binding (12). Increased blood levels of CS proteins and activation products such as C3, soluble C5b-9 (sC5b-9), and mannan-binding lectin (MBL) have been observed in diabetes patients and shown to be positively associated with DR (8,13–19). Local complement activation in the retina and surrounding tissue that is seen in DR includes increased levels of C3, C3d, and MAC deposition (20,21), as well as of C5a and complement factor I (CFI) (22–24).

In glaucoma, it has been suggested that an early inflammatory response (25,26) and increased intraocular pressure cause a local ischemia at the optic nerve head (27,28). The ischemic ganglion cells bind C1q, CS becomes activated and deposited (as C5b-9), and ganglion cells become compromised (27–29). In addition, the glaucomatous ganglion cells display low amounts of complement factor H (CFH), further dysregulating the CS (28).

AU (partial or complete inflammation of the uvea) is assumed to be the consequence of a defect in ocular immune privilege and a dysregulated complement system. Inflammatory leukocytes (with no known trigger) invade the retinal tissue and contribute to the breakdown of the aqueous–blood barrier as well as the blood–retinal barrier (30,31). Both complement activation products such as C3a, C3c, C3d, C4c, C5a, and autoantibodies against ocular proteins have been detected in the aqueous humor from patients with AU as well as in idiopathic uveitis (32–34).

AMD, the most investigated of the aforementioned diseases, is a complex progressive neurodegenerative disease in which the macula is principally affected (35). AMD is associated with a number of factors, including age, CS dysregulation, genetic factors (polymorphism in diverse CS genes), oxidative stress, and sunlight (6,36–40). Submacular drusen (41,42) between the retinal pigment epithelium (RPE) and the underlying Bruch’s membrane primarily affect the post-mitotic RPE cells, which are essential for photoreceptor viability (43). Progressed AMD is categorized as either geographic atrophy (GA) or neovascular, or exudative, AMD. GA is characterized by a loss of RPE, photoreceptors, and choriocapillaries; neovascular AMD is characterized by abnormal choroidal vessel growth into the retinal space (41). While much is known about the disease-related attributes of AMD, it is still unclear what pathological processes initiate drusen accumulation and the accompanying degeneration of the retinal cells.

Several lines of evidence, however, indicate that the CS plays a critical role in AMD pathogenesis (6,44–46). Genetic variations in several human complement components and regulators, such as C2, C3, CFH, CFI, and factor B, have all been correlated with the occurrence of AMD (6,46–51). Also, abundant complement-activating molecules and complement proteins, regulators, and receptors have been found to be expressed in drusen, the retina, and underlying retinal tissue, including CRP, immunoglobulin, C1q, C3, iC3b, C3a, C5a, sC5b-9, CFB, CFD, CFH, FHL-1, CD46, CD55, and CD59 (6). Soluble factors such as C3a, C5a, and sC5b-9 have been found in the plasma of AMD patients (52,53). A recent report by Schick and co-workers has indicated that the anaphylotoxin C3a and sC5b-9 are present in the aqueous humor of AMD patients (54), suggesting a local as well as a systemic complement activation in AMD. It has been postulated that photo-oxidatively damaged RPE cells directly activate different complement components (42,55,56).

Given their high metabolic rate, RPE cells are highly are susceptible to oxidative stress and associated retinal inflammation (57–59). In an effort to preserve homeostasis, recruited inflammatory cells (e.g. macrophages and microglial cells) differentiate into active phagocytic, migrating cells that release proinflammatory mediators (6,58,60–62), fueling the complement-mediated inflammatory process seen in AMD. However, AMD pathogenesis has largely remained unclear, and the cellular interactions leading to CS activation in AMD are not yet identified and are therefore in need of further investigation.

Although there is convincing evidence of CS involvement in AMD, DR, glaucoma, and uveitis, there is still an absence of complement-based treatment opportunities. Unfortunately, further research has been hampered by a lack of good animal models for these diseases. The neovascularization in AMD can be controlled by intravitreal antiangiogenic therapy (63,64), but treatment opportunities for the most common forms of AMD (submacular drusen and GA) remain limited. Hence, there is an urgent need for new treatments and innovative strategies to incorporate the diverse CS proteins that appear to be promising targets for treatments.

Here we describe a new co-culturing system, based on porcine retina and human RPE cells, that can be used to mimic a diverse set of eye diseases. By using light-exposed post-confluent spontaneously arising human retinal pigment epithelial (ARPE) cells, mimicking an old RPE cell layer (65) in apical contact with the external parts of the adult porcine retina and co-culturing them on a porous support, we have designed a model for experimental retinal degeneration. This system allows the investigation of diverse proteins as well as interactions between tissues and cells and is based on the use of cultured ARPE cells to investigate the degenerative process as a well-established and defined system (66). Our system has allowed us to conduct a focused investigation of the role of the CS within a controlled experimental environment.

Retinal microglial cells and RPE cells appeared to be the major complement producers in this *in vitro* culture system. A diverse set of complement proteins, regulators, CS receptors, as well as anaphylatoxin activation fragments was found after co-culture. Hence, we believe that this co-culture system can serve as a useful model for experimental retinal neurodegenerative diseases and investigations of CS involvement.

## Method and materials

### Tissue preparation of porcine retinal explants

The Swedish Board of Agriculture approved the experimental procedures (Sweden, Jönköping ref. 6.7.18-4833/15). Adult porcine eyes were collected from the local abattoir and transported to the laboratory in cold CO_2_-independent media (Gibco, Life Technologies, Carlsbad, CA). After a rapid spray with ethanol, the cornea, lens, and vitreous body were removed. Cone-enriched (67,68) neural retinal explants of about 10 mm^2^ were punched out. Non-cultured control retinas were taken from directly immersion-fixed eyes.

### Feeder layers using the ARPE-19 cell line

**Figure 1.**
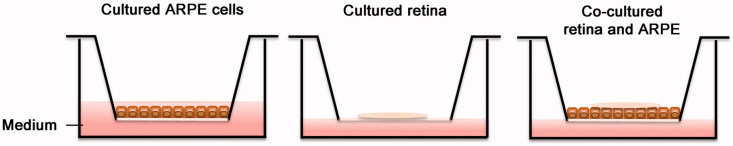
Culture system setup: ARPE cells cultured on transwell inserts for 5 weeks post-confluence. Retinas were cultured on inserts with the photoreceptor cells facing down, with or without post-confluent ARPE cells. All cultures were exposed to cyclic light for 8 h/day for 3 or 5 days *in vitro*.

The spontaneously arising human retinal pigment epithelium cell line, ARPE-19 cells (160,000 cells) were seeded onto Millicell^®^-PCF 0.4-μm culture plate inserts (Millipore, Bedford, MA, USA) (Figure 1). Preliminary experiments were performed to find the co-culture setup generating minimal cell death over time for retinal cultures (data not shown). Cells were either cultured to sub-confluence (for real-time polymerase chain reaction [PCR] measurements) in 10% fetal calf serum in Dulbecco’s modified Eagle’s medium/F12 (DMEM/F12) (Gibco) or allowed to grow for 5 weeks post-confluence to mimic an aged RPE cell layer. Passages 6–12 were used. The ARPE-19 cells proliferated in a medium composed of DMEM/F12 supplemented with 2% B27 supplement (Gibco), 1% N2 supplement (Gibco), 1% penicillin/streptomycin, and 2 mM glutamine (Sigma-Aldrich, St Louis, MO, USA), and the medium was changed every third day.

### Co-culture of porcine retinal explants with or without ARPE-19 feeder layers

Retinal explants of about 10 mm^2^ were divided into four equal sections and explanted on culture plate inserts, with or without post-confluent ARPE-19 cells (Figure 1). Retinas were placed with the vitreal side oriented upwards and the photoreceptors oriented downwards. One or two retinal sections were explanted onto each transwell insert and placed in multiwell culture dishes containing 1.2 mL of culture medium per well. A droplet of cell culture medium was placed on the retinas, covering them with a moist film. Cultures were exposed to cyclic light and dark illumination for 3 or 5 days *in vitro* at 37 °C with 95% humidity and 5% CO_2_, as described previously by Mohlin and co-workers (69). In brief, cultures were illuminated by warm white light for 8 h/day with an illuminance of 80 lux. Cell culture medium was exchanged every day, then frozen (–80 °C) for further analysis.

### Transmission electron microscopy

Specimens were fixed with 2% paraformaldehyde (Sigma-Aldrich, St Louis, MO, USA) and 2% glutaraldehyde (Agar Scientific Ltd, Stansted, United Kingdom) in 0.1 M phosphate-buffered saline (PBS), pH 7.4, overnight at 4 °C. The fixation was followed by repeated rinsing in cacodylate buffer (Sigma-Aldrich), after which samples were post-fixed in 1% osmium tetroxide (Agar Scientific). The specimens were dehydrated using increasing concentrations of ethanol, and embedded in Epon resin (Sigma-Aldrich), and semi-thin sections were obtained and examined. Ultrathin sections (50 nm) were taken from selected areas and counterstained with uranyl acetate (Ted Pella, Inc., CA, USA) and lead nitrate (BDH Middle East LLC, Dubai, UAE), and examined using a JEOL 1230 transmission electron microscope (Leol, Tokyo, Japan). Data associated with transmission electron microscopy (TEM) can be found as supplementary material.

### TUNEL assay

In order to detect neuronal cell death, a commercial terminal deoxynucleotidyl transferase (TdT)-mediated dUTP nick-end labeling (TUNEL) fluorescein *in situ* cell death detection kit (Roche, Mannheim, Germany) was applied to retinal sections according to the manufacturer’s instructions. In brief, sections were treated with TUNEL for 45 min at 37 °C in darkness; after several washes, the sections were mounted with Vectashield anti-fading mounting medium containing 4',6-diamidino-2-phenylindole dihydrochloride (DAPI). The number of TUNEL-labeled cells/mm^2^ in the inner and outer nuclear layers (INL and ONL) was counted manually by using a Nikon epifluorescence microscope (Nikon, Tokyo, Japan), and images were captured with a digital camera acquisition system (Nikon, DS-U1) using Nis-element imaging software, version 4.3, from Nikon.

### Immunohistochemical staining

After culture, specimens (post-confluent ARPE-19 cells alone, co-cultures of adult porcine retinas and ARPE-19 cells, and control retinas cultured on Millicell^®^-PCF 0.4-μm culture plate inserts) were fixed by immersion in 4% paraformaldehyde (Apoteket, Gothenburg, Sweden) overnight at 4 °C. After several rinses with 0.1 M Sorensen’s phosphate buffer (Sigma), pH 7.2, the samples were cryoprotected in increasing concentrations of sucrose (10%–25%) (Sigma-Aldrich) in 0.1 M Sorensen’s phosphate buffer (Sigma), pH 7.2. The tissue samples were embedded in Cryomount^TM^ (HistoLab, Gothenburg, Sweden), cryosectioned at 10 μm, and stored at −20 °C. Uncultured normal retinas were directly fixed and cryosectioned in a similar fashion. Primary antibody (Table 1) was applied to the sections and incubated overnight at 4 °C. After being rinsed in phosphate-buffered saline (PBS) (Medicago, Uppsala, Sweden), the sections were treated with selected fluorescent secondary antibodies: fluorescein isothiocyanate (FITC)-conjugated goat anti-mouse IgG Fab (1:500, Jackson Laboratories, West Grove, PA, USA), Alexa Fluor 488-conjugated donkey anti-rabbit IgG Fab (1:500, Jackson Laboratories), or Alexa Fluor 568-conjugated goat anti-mouse IgG Fab (1:500, Life Technologies) and incubated at room temperature for 45 min in darkness. All antibodies were diluted in the blocking solution (1% BSA in PBS). Finally, the sections were rinsed with PBS and counterstained using a 4',6-diamidino-2-phenylindole (DAPI)-containing mounting medium (Vector Laboratories, Burlingame, CA, USA). Exclusion of the primary antibody was used as a negative control in all experiments.

**Table 1.** Primary antibodies used in immunohistochemical analysis.

Antibody	Source	Company	Working dilution	Cell specificity
Retinal proteins				
Calbindin	Rabbit polyclonal	Synaptic Systems, GmbH, Göttingen, Germany	1:2000	Cone photoreceptors, bipolar, horizontal, amacrine and ganglion cells
Glial fibrillary acidic protein (GFAP)	Rabbit polyclonal	ProteinTech Group, Chicago, USA	1:2000	Astrocytes and Müller glial cells
Ionized calcium binding adaptor molecule 1 (IBA 1)	Rabbit polyclonal	ProteinTech Group	1:500	Resident and active microglial cells
Retinal pigment epithelium 65 (RPE65)	Mouse monoclonal	Merck Millipore, CA, USA	1:500	RPE cells
Zonula occludens 1 (ZO-1)	Rabbit polyclonal	Bioss, MA, USA	1:500	Tight junctions
Complement proteins				
Complement component 1q (C1q)	Rabbit polyclonal	Dako, Glostrup, Denmark	1:500	C1q
C3c	Rabbit polyclonal	Dako	1:1000	C3
Soluble C5b-9 (sC5b-9)	Mouse monoclonal	Diatec Monoclonals AS, Oslo, Norway	1:200	sC5b-9
Complement regulators				
Complement factor H (CFH)	Mouse monoclonal	Hycult Biotech	1:500	CFH
CFI	Sheep polyclonal	The Binding Site Inc., San Diego CA, USA	1:500	CFI
Complement receptors				
C3a receptor (C3aR)	Mouse monoclonal	Hycult Biotech	1:400	C3aR
C5aR	Mouse monoclonal	Hycult Biotech	1:400	C5aR
C5aR-like 2 (C5L2)	Mouse monoclonal	Biolegend Inc., CA, USA	1:400	C5L2

### Detection of fluid-phase proteins by Luminex xMAP

To determine whether complement proteins were secreted from the various samples (ARPE cells, retinal explants, and co-cultures), we analyzed conditioned medium collected during the culture process. The medium was frozen and stored at –80 °C until measurements were made in a Bio-Plex^®^ MAGPIX multiplex reader (Bio-Rad Laboratories Inc., CA, USA). In brief, conditioned media from the aforementioned samples were examined for the presence of the secreted CS proteins C1q and C3 and for the CS regulators C4BP, CFH, and CFI, using the antibodies shown in Table 2. It was confirmed by dot blot that these antibodies, which were raised against human complement proteins, also recognize the porcine counterparts (data not shown). The assays were performed in 96-well flat-bottomed microtiter plates (Bio-Plex Pro, Bio-Rad), and antibody-coupled beads were used at 2,500 beads per well. All standards, controls, and samples were tested in duplicate. Conditioned medium (50 μL) from each sample was added to the coated beads and incubated for 30 min; the beads were then washed in washing buffer, and the appropriate biotinylated antibody was added (Table 2). Unbound antibodies were washed away, and the secreted bound proteins were detected by adding streptavidin-phycoerythrin at a 1:100 dilution (Bio-Rad). The secreted complement proteins were quantitated in a Multiplex Reader (Bio-Rad) as mean fluorescence intensity (MFI), using Bio-Plex manager MP Software and Bio-Plex Manager 6.1 (Bio-Rad) to calculate the results.

**Table 2.** Details of antibodies used in Luminex measurements.

Specificity	Source	Utility	Company	Working dilution/bead
C1q	Mouse monoclonal	Capture	Hycult	3 μg/1.25*10^6^ beads
C1q	Mouse monoclonal	Detection^a^	Hycult	1 μg/mL
C3c	Rabbit polyclonal	Capture	Dako	3 μg/1.25*10^6^ beads
C3c	Rabbit polyclonal	Detection^a^	Dako	0.5 μg/mL
C4BP	Mouse monoclonal	Capture	Bio-Rad	3 μg/1.25*10^6^ beads
C4BP	Mouse monoclonal	Detection^a^	Bio-Rad	1 μg/mL
Factor H	Mouse monoclonal	Capture	Hycult	3 μg/1.25*10^6^ beads
Factor H	Mouse monoclonal	Detection^a^	Hycult	4 μg/mL
Factor I	Mouse monoclonal	Capture	Abcam	1.5 μg/1.25*10^6^ beads
Factor I	Rabbit polyclonal	Detection^a^	Abcam	3.3 μg/mL

a Biotinylated antibody.

### RNA extraction and quantitative real-time PCR

Extraction of mRNA from ARPE cells (non-cultured control ARPE cells, post-confluent control ARPE cells, and co-cultured ARPE cells) was performed using an RNeasy Minikit (Qiagen Inc., Valencia, CA, USA). In brief, retinal explants were lysed in RLT buffer supplemented with 10 μL/mL mercaptoethanol (Sigma-Aldrich) and homogenized. Each sample was loaded onto a silica column, followed by washing and elution in RNase-free water according to the manufacturer’s instructions. Total RNA from retinas (1 μg) was converted to cDNA using oligo-dT16 primers (1 μM) (Applied Biosystems, Foster City, CA, USA) and the Omniscript RT kit (Qiagen). RNA was controlled for genomic DNA contamination. The primers (Life Technologies) used are summarized in Table 3. Quantitative real-time PCR (qRT-PCR) data were collected using the Applied Biosystems 7500 Real-Time PCR system (Applied Biosystems) with specific primers (Life Technologies) (Table 3) and the SYBR Green PCR Master Mix detection method on triplicates of the cDNA samples. The following thermal conditions were used: 50 °C for 2 min and 95 °C for 10 min, 40 cycles of 95 °C for 15 s, and 60 °C for 1 min, including a final melting curve step. The *Ct* values obtained were normalized to the housekeeping reference gene 18S for the human ARPE cells (Table 3).

**Table 3.** Target-specific primers for factor H, factor I, and 18S.

Gene	Primers (forward and reverse)	Size (bp)	Reference sequence
Factor H	5′-AAC AGA TTG TCT CAG TTT ACC TAG C-3′ 5′-ACC CGC CTT ATA CAC ATC C-3′	76	NM_000186
Factor I	5′-GAA GTT GGC TGT GCA GGC TT-3′ 5′-CTG CAT CCA TGT CAG CAG TC-3′	73	NM_000204
18S	5′-ATC CCT GAA AAG TTC CAG CA-3′ 5′-CCC TCT TTG GTG AGG TCA ATG-3′	155	NM_022551

### Detection of fluid-phase complement activation products by ELISA

Conditioned media from the various samples were investigated for fluid-phase anaphylotoxin C3a and for sC5b-9 by sandwich ELISA. The amount of generated C3a was measured with monoclonal antibody (mAB) anti-C3a 4SD17.3 as the capture antibody and polyclonal biotinylated anti-C3a (70). Detection of sC5b-9 complexes was made using mAB anti-neoC9 aEII (Diatec Monoclonals AS, Oslo, Norway) as the capture antibody (71) and detected with a sheep polyclonal anti-C5 antibody (Acris Antibodies GmbH, Herford, Germany). The abovementioned complexes were detected with HRP-conjugated streptavidin (GE Healthcare, Uppsala, Sweden). Zymosan-activated serum served as a standard for the analysis, and pooled diluted zymosan-activated serum from blood donors was used as a control.

### Microscopy and data analysis

Labeled sections were examined in an epifluorescence microscope (Nikon, Tokyo, Japan) equipped with appropriate filters. Images were captured with a digital acquisition system (DS-U1, Optronics) and analyzed using the software Nis elements 4.30 (Nikon). Adobe Photoshop CS4 11.0 (Adobe, San Francisco, CA, USA) was used for image brightness/contrast adjustments. The individual fluorescent images were all treated in the same way, and no information was added. Counts of TUNEL-labeled cells were made on four areas per section on two non-consecutive sections, and measurements were averaged to a single value for each labeled retina. Data are expressed as mean values ± standard error of the mean (mean ± SEM). Data were assumed to be normally distributed, and paired Student’s *t* tests or ANOVA followed by Tukey’s multiple comparison *post hoc* tests were used for statistical comparisons. *P* ≤ 0.05 was considered significant. Statistical analyses were performed in GraphPad Prism 7a software (La Jolla, CA, USA).

## Results

### Expression of native RPE cell markers in post-confluent ARPE cells

**Figure 2.**
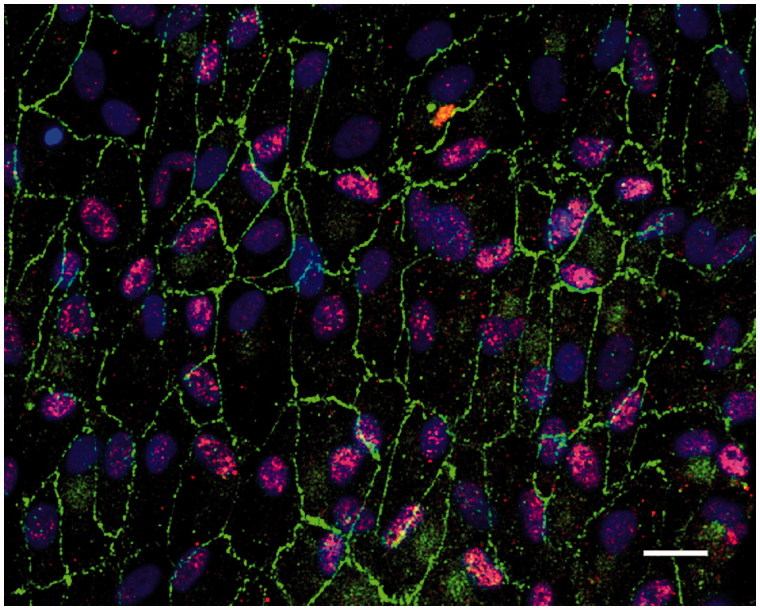
Immunofluorescent images of human post-confluent ARPE cells double-labeled for the tight junction protein ZO-1 (green) and RPE65 (red, specific for RPE cells) and counterstained with DAPI (blue). Scale bar, 20 μM.

Under the culture conditions used here, the post-confluent and light-exposed ARPE cells displayed characteristics of native markers (Figure 2). The differentiated ARPE cells showed monolayer formation (with some cells occasionally producing several layers) and a hexagonal shape. The tight junction-associated protein zonula occludens-1 (ZO-1) was restricted to the cortical areas at the cell edges (Figure 2), indicating a tight junction organization typical of native RPE. RPE65 was also expressed in the post-confluent ARPE-cells (Figure 2).

### Detection of cell death by TUNEL assay

**Figure 3.**
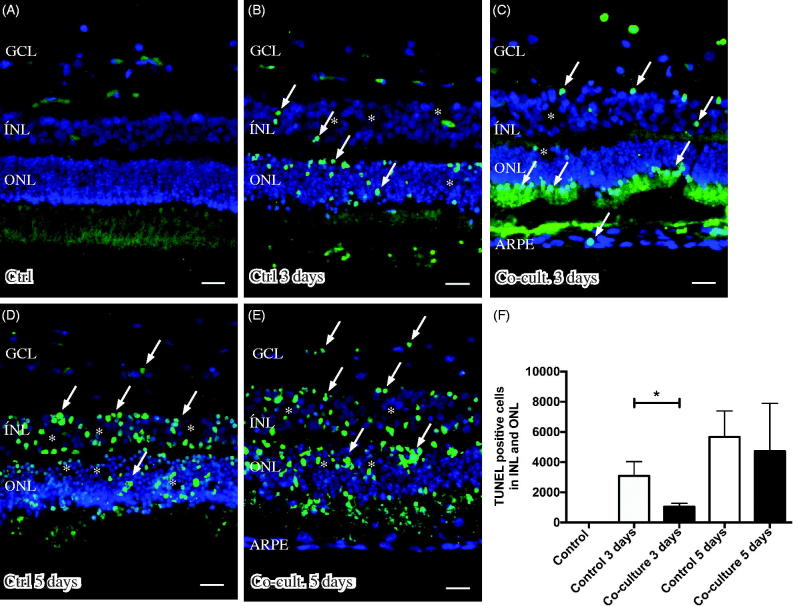
Immunofluorescent images and analysis of cell death by TUNEL assay (green); the cell nuclei are counterstained with DAPI (blue) in non-cultured control retinas (A) and retinas cultured or co-cultured for 3 (B, C) or 5 days *in vitro* (D, E). Cell death in the outer nuclear layer (ONL) and in the inner nuclear layer (INL) increased over time in the cultured (indicated by arrows in B and D) and co-cultured (indicated by arrows in C and E) retinas when compared to the non-cultured control retinas (A). After 3 days of co-culture with ARPE cells (C, F), cell death was decreased in the retinal specimens when compared to the control retinas (B, F) after 3 days of culture. Cultured retinas retained normal layering post-culture (B–E), shown by separate nuclear layers in the DAPI-stained sections: the outer nuclear layer (ONL), inner nuclear layer (INL), and inner ganglion cell layer (GCL). After culture, the specimens showed holes and injuries, indicated by (*) in the retinal tissue. Occasionally, some TUNEL-labeled ARPE cells were found in the co-culture setup (arrow in ARPE layer in C). Data are expressed as means ± SEM; *P < 0.05; *n* = 4; scale bars, 20 μM.

Retinal tissue layering was maintained after culture (Figure 3(B–E)) when compared to non-cultured control retinas (Figure 3(A)), as investigated by DAPI staining. The nuclear layers of the retina showed a normal distribution and were separated into a ganglion cell layer (GCL) as well as an inner and outer nuclear layer (INL and ONL) (Figure 3(A–E)). The height of the nuclear layers decreased *in vitro* (Figure 3(B–E)), a known feature related to cell death and retinal remodeling (69). After co-culture of the retinas with ARPE cells, the retinal sheet integrity was preserved, as indicated by decreased cell death (1047 ± 136 dead cells) in the retina co-cultured for 3 days (Figure 3(C,F)) as compared to retinas cultured without ARPE cells (3085 ± 549 dead cells) for 3 days (Figure 3(B,F)). The effect on nuclear layers was not observed after 5 days (Figure 3(D–F)). The non-cultured control retinas did not show any cell death, as indicated by TUNEL staining (Figure 3(A)). Co-culturing retinas with 160,000 ARPE cells was found to be optimal for investigating cell death (in preliminary experiments, retinas were co-cultured with 10,000, 60,000 [data not shown], or 160,000 ARPE cells).

### Immunohistochemical staining of complement proteins

**Figure 4.**
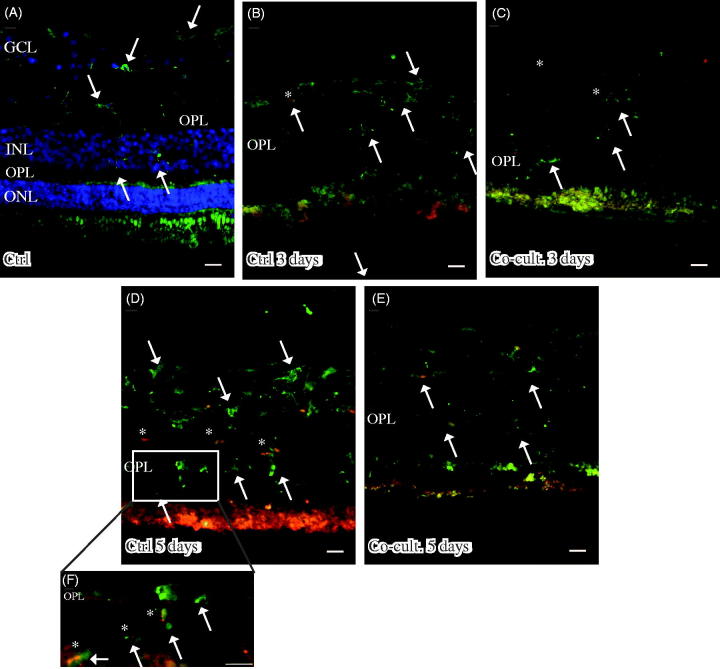
Immunofluorescent staining for C1q (red) and the microglial marker ionized calcium binding adaptor molecule 1 (IBA-1, green) in retinal tissue and ARPE cells. The non-cultured control retina (A) is counterstained with DAPI, and the nuclear layers are indicated: outer, inner, and inner ganglion cell layer (ONL, INL, and GCL), respectively, as well as the outer plexiform layer (OPL). C1q is not shown in non-cultured control retinal cells (A). Resident microglial cells (A) located in the inner retinal tissue (indicated by arrows) and microglial protrusions extend all the way to the OPL (indicated by arrows in the OPL) in the non-cultured control tissue. After 3 days in culture the migrating and active amoeboid-like microglial cells (indicated by arrows) become sparsely C1q-expressing (indicated by *). After 5 days in culture, active microglial cells start to migrate even more, through the OPL and into the outer retinal tissue (D–F). Active microglial cells in retinal tissue cultured for 5 days (D and F) are double-labeled with anti-C1q. There seem to be more active and C1q-positive microglial cells (indicated by *) in the cultured specimens (D, F) than in the co-cultured specimens (E) after 5 days. Scale bars, 20 μM.

Immunofluorescent staining for C1q was seen in cultured specimens (Figure 4(B–F)). C1q expression was found in the cultured control retinas and in the co-cultured specimens after 5 days (Figure 4(D–F)), although the co-cultured specimens (Figure 4(E)) appeared to express less C1q than did the cultured control retinas (Figure 4(D)). No staining of C1q was ever found in the non-cultured control retinal cells (Figure 4(A)), although sparse labeling of C1q was seen in the blood vessels in these non-cultured controls (data not shown). Inflammatory active and migrating microglial cells are a known feature of retinal degenerative disorders (6,58,60–62,72), and microglial cells have been connected to C1q expression (73). The staining pattern we saw for C1q therefore prompted us to carry out double staining with the microglial cell marker IBA1. The staining pattern indicated that C1q was co-localized in a punctate-like manner with scattered migrating microglial cells in the cultured specimens (Figure 4(B–F)). C1q labeling was even more pronounced after 5 days (Figure 4(D–F)), and the control retina cultured for 5 days seemed to express more C1q than did the co-cultured retinas; in addition, the staining in the control retina coincided more frequently with the migrating microglial cells (Figure 4(D–F)). Sparse C1q labeling was also seen in the ganglion cell layer after 5 days (Figure 4(D,E)).

**Figure 5.**
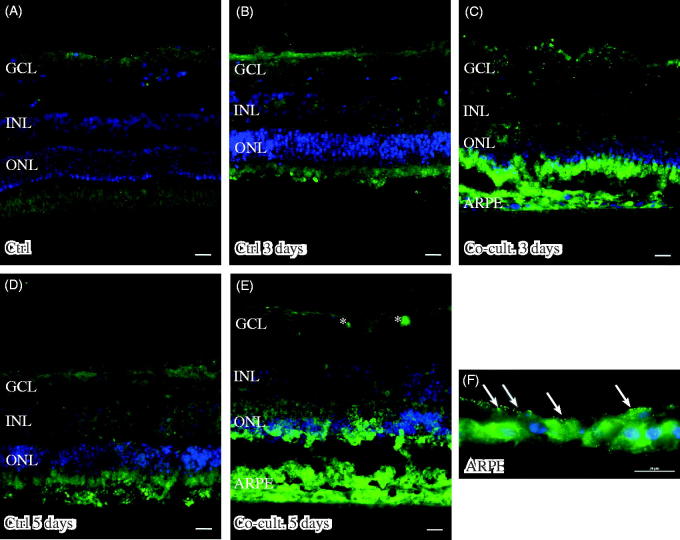
Immunofluorescent staining for complement protein 3 (green) in retinal tissue and ARPE cells; nuclei are counterstained with DAPI. Retinal nuclear layers (ONL, INL, and GCL) are indicated. C3-positive labeling is shown in the ARPE cells (C, E, F) and in the outer retina in co-cultured specimens (C, E). Sparse C3-positive ganglion cells are also seen after 5 days of co-culture (*). C3 labeling in the cultured ARPE cells is cytoplasmic and punctate-like, toward the apical portion of the ARPE cells (indicated by arrows in F). Scale bars, 20 μM.

Retinas labeled with the anti-C3 antibody showed C3 expression in the outer part of the outer nuclear layer (ONL) in the co-cultured retinas (Figure 5(C,E)). The ARPE cells showed a punctate staining for C3 after being cultured alone (Figure 5(F)) or with porcine retinas (Figure 5(C,E)). C3 labeling was mainly restricted to the apical part of the ARPE, toward the overlying retina. C3 expression was not seen in either the non-cultured (Figure 5(A)) or cultured control retinas (Figure 5(B,D)). C3-positive staining was also seen in the ganglion cell layer in the co-cultured specimens (Figure 5(C,E)).

**Figure 6.**
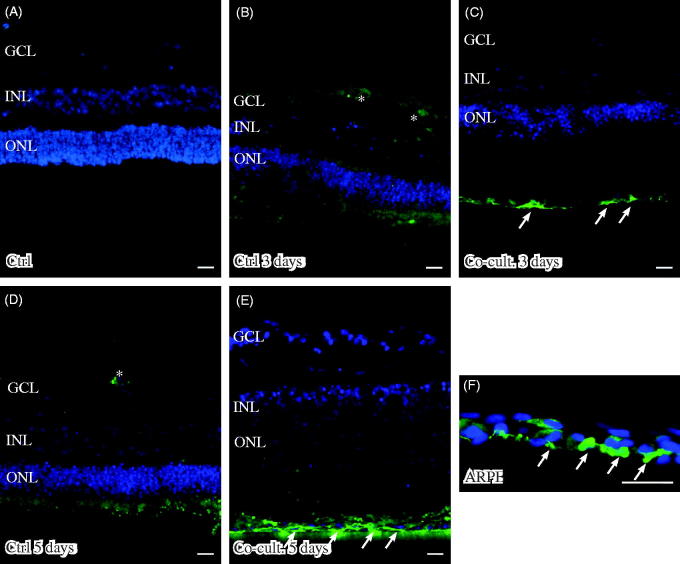
Immunofluorescent staining for complement protein 4 (green) in retinal tissue and ARPE cells; nuclei are counterstained with DAPI. Retinal nuclear layers (ONL, INL, and GCL) are indicated. C4-positive labeling is seen only in the ARPE cells (C, E, F). C4 labeling in the cultured ARPE cells is cytoplasmic, and C4 shows a more intense expression on the basal part of the ARPE cells (C, E, F) (indicated by arrows in F). Sparse C3-positive ganglion cells are obvious in the cultured control retinal tissue after 3 and 5 days of culture (indicated by * in the GCL). Scale bars, 20 μM.

Immunostaining of C4 showed labeling in the ARPE cell layer, when the ARPE cells were either cultured alone (Figure 6(F)) or co-cultured for 3 (Figure 6(C)) or 5 days (Figure 6(E)). The expression of C4 seemed to be more intense in the basal portion of the ARPE cells (Figure 6(C,E)) than toward the outer part of the retina. Neither non-cultured control retinas (Figure 6(A)) nor the cultured control retinas showed any expression of C4 (Figure 6(B,D)). Neither of the two regulators, CFH and CFI, showed any positive immunolabeling in the retinal tissue or the ARPE cells, in either the controls or the co-cultured specimens (data not shown).

**Figure 7.**
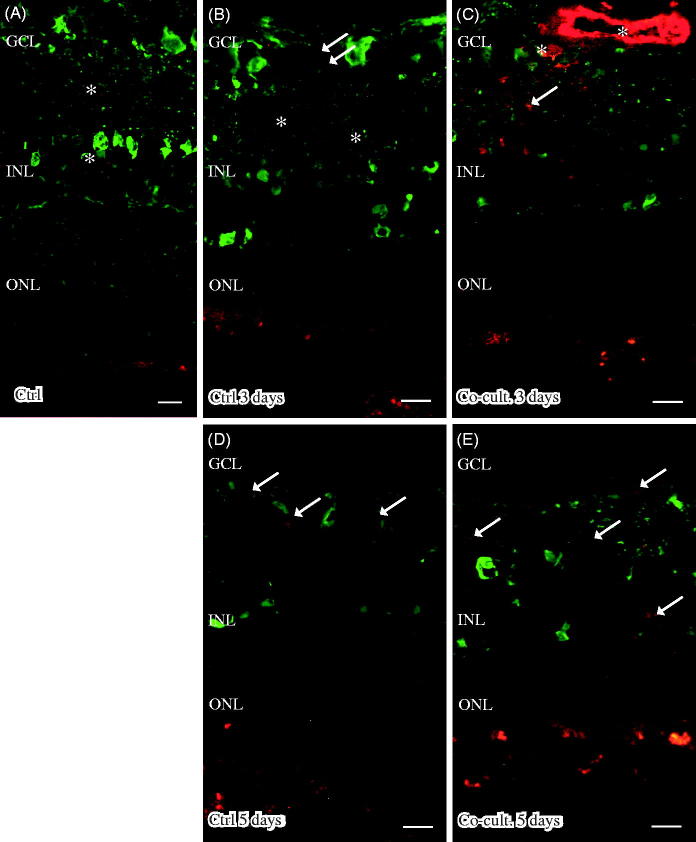
Immunofluorescent staining for calbindin (green) and the soluble terminal complement complex (sC5b-9, red) in retinal tissue and ARPE cells. Retinal nuclear layers (ONL, INL, and GCL) are indicated. Anti-calbindin antibody labels ganglion, horizontal, and amacrine cells in the retinal tissue (A–E). sC5b-9 (indicated by *) labels blood vessels in non-cultured control retinas (A), in retinas cultured for 3 days (B), and in retinas co-cultured for 3 days (C). After retinal culture or co-culture for 3 or 5 days, ganglion cells are sparsely positive for sC5b-9 (indicated by arrows in B–E). In co-cultured specimens, sC5b-9 seems to coincide with calbindin-positive cells in the INL. Scale bars, 20 μM.

Immunolabeling of the retinas and cells for the terminal complement complex sC5b-9 was seen in the inner retinal blood vessels and Bruch’s membrane in the non-cultured controls (Figure 7(A)). Inner retinal blood vessels were also labeled in the retinas co-cultured for 3 days (Figure 7(B) and (C)). After 5 days, the abundant blood vessels in the inner retina were absent, as was sC5-9 immunoreactivity. However, a weak staining of sC5b-9-positive ganglion cells, co-labeled with calbindin, was seen after 5 days (Figure 7(B–E)). Sparse sC5b-9-positive labeling was also seen in the INL in co-cultured specimens (Figure 7(C,E)).

**Figure 8.**
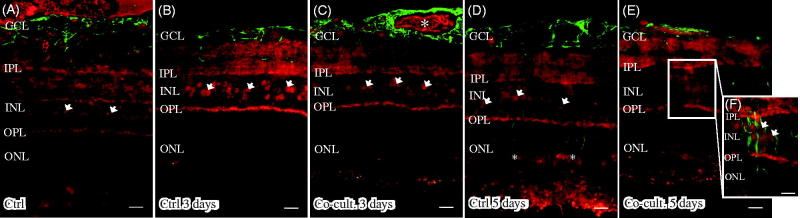
Immunofluorescent staining for glial fibrillary acidic protein (GFAP; green) and the complement anaphylatoxin receptor C5aR (red) in retinal tissue and ARPE cells. Retinal nuclear layers (ONL, INL, and GCL) are indicated. C5aR shows endogenous expression in the retinal tissue, in the inner retina GCL, INL, and the plexiform layers (IPL and OPL). C5aR (indicated by arrowheads) labels Müller cell nuclei (A–D, F). GFAP labels astrocytes (A–E) and major Müller cells in the retinal tissue (A–F). In the cultured gliotic retinas, Müller cells show increased GFAP staining that is more intense after 5 days (D), and GFAP is expressed up to the Müller cells’ distal processes, stretching to the external limiting membrane in the outer retina (indicated by *). Also, GFAP-positive filaments surround the C5aR-positive Müller cell nuclei in the INL (E and F). Scale bars, 20 μM.

Samples were also examined to detect the expression of the C3a and C5a receptors (R) C3aR, C5aR, and C5L2 (Figure 8(A)). Neither C3aR nor C5L2 could be detected in retinal cells or tissue (data not shown). However, C5aR was found to be endogenously expressed in blood vessels, the ganglion cell layer (GCL), the inner plexiform layer (IPL), in Müller cell nuclei in the inner nuclear layer (INL). C5aR has been reported to be constitutively expressed in retinal tissue (6,74) and in human primary Müller cells (75); these findings were confirmed here in the porcine retinas (Figure 8(A)). The expression of C5aR in Müller cell nuclei is surrounded by the gliotic Müller cell body (Figure 8(B–F)).

### Detection of fluid-phase proteins by Luminex xMAP

**Figure 9.**
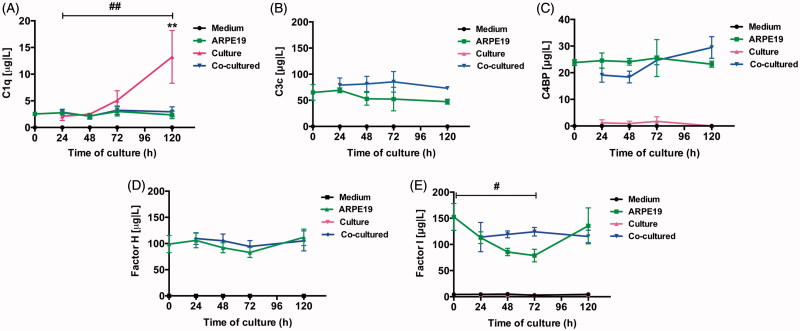
Luminex analysis of complement components (C1q, C3, C4BP, CFH, and CFI) in conditioned media from post-confluent ARPE cells (green line) and cultured retinas (pink line) as well as co-cultured retinas (blue line), cultured for 0–5 days. Cell culture medium (black line) was also included; none of the investigated complement components was found in this medium. C1q secretion from retinal cultures cultured for 5 days (A) was higher than that from ARPE cells cultured alone or ARPE cells co-cultured with porcine retinas. C3 was secreted from ARPE cells and from co-cultures (B). The regulators C4BP (C), CFH (D), and CFI (E) were all secreted from the ARPE cells and the co-cultures, but none of the regulators was secreted in any major amount from the cultured control retinas. There was a temporal decrease in the CFI secretion after 3 days (E) when compared to the co-cultured specimens. Data are expressed as means ± SEM; *P < 0.05; **P < 0.01; *n* = 4.

Measurement of secreted complement proteins was performed on conditioned medium taken from post-confluent ARPE cells and from cultured and co-cultured retinas. C1q was found in the conditioned medium taken from retinas cultured for 3 or 5 days (Figure 9(A)), and a significant increase in C1q (from baseline to 13.3 ± 5.0 μg/L) was seen after 5 days. No large quantities of C1q were found to be secreted from the ARPE cells or from the co-cultured specimens (Figure 9(A)). Collected media taken from ARPE cells and co-cultured retinas showed secreted C3 during culture; in contrast, no secretion of C3 was seen in the cell culture medium taken from the cultured control retinas (Figure 9(B)). The complement protein regulators C4BP (Figure 9(C)), CFH (Figure 9(D)), and CFI (Figure 9(E)) were secreted from the post-confluent ARPE cells as well as from the co-cultured specimens (Figure 9(C–E)). There was a temporal decrease in ARPE CFI secretion after 3 days when compared to the co-cultured specimens; although minor, this decrease was significant (Figure 9(E)). None of the regulators was secreted in any large quantity from the cultured control retinas, and none of the complement proteins was found at any time in the cell culture medium.

### RNA extraction and quantitative real-time PCR

**Figure 10.**
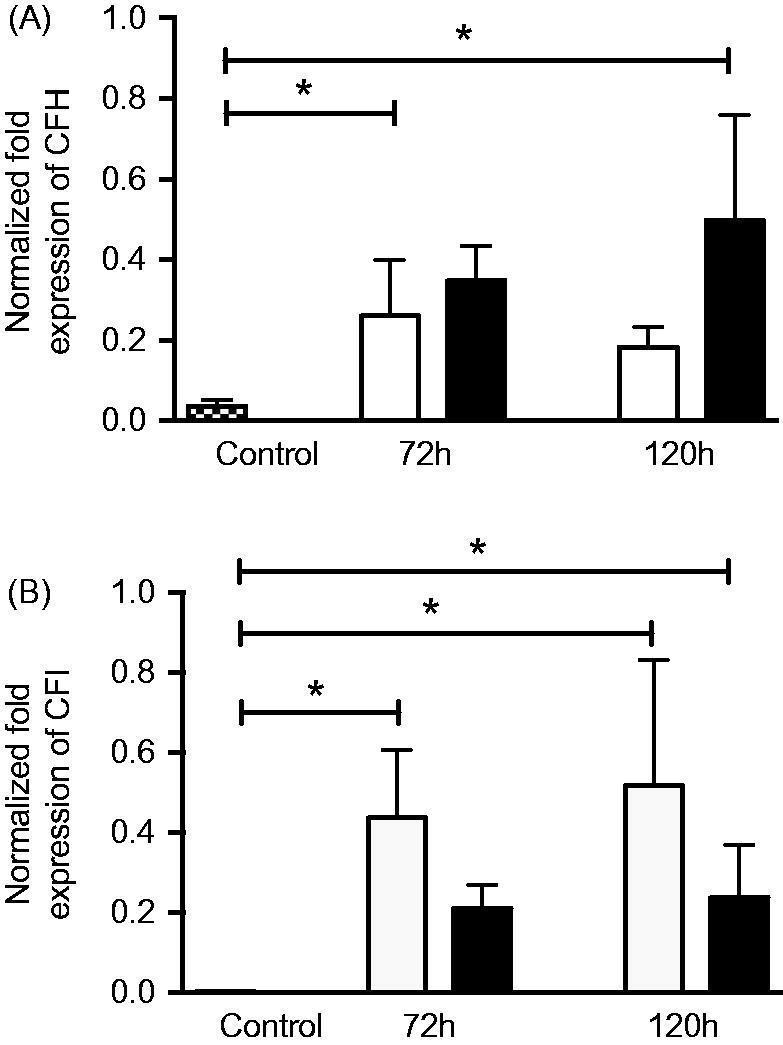
Real-time quantitative PCR analysis of the alternative pathway regulators (A) complement factor H (*CFH*) and (B) complement factor I (*CFI*): normalized Ct values in young control ARPE cells (dashed bars), post-confluent cultured ARPE cells (white bars), and post-confluent co-cultured ARPE cells (black bars). After 3 days, there was a significant increase in *CFH* mRNA expression in the post-confluent ARPE cells as compared to the young cultured ARPE cells. After 5 days, the co-cultured ARPE cells showed an increase in *CFH* mRNA expression when compared to the control cells. *CFI* was significantly increased in post-confluent ARPE cells cultured for an additional 3 or 5 days when compared to the young control ARPE. The amount of *CFI* mRNA in co-cultured ARPE cells was significantly increased after 5 days in culture when compared to the control values. Data are expressed as means ± SEM; *P < 0.05; *n* = 4).

The mRNA expression of the alternative pathway complement regulators *CFH* (Figure 10(A)) and *CFI* (Figure 10(B)) was significantly upregulated in post-confluent ARPE cells allowed to grow for 3 or 5 days alone or in co-culture, when compared to ARPE control cells that were sub-confluent and grown in a serum-rich environment. The aged, post-confluent ARPE cells grown in serum-free medium showed a 7-fold increased expression of *CFH* (0.26 ± 0.1-fold change) (Figure 10(A)) after culture for an additional 3 days post-confluence when compared to the control (0.04 ± 0.0-fold change). Co-cultured ARPE cells at 5 days post-confluence (0.50 ± 0.2-fold change) showed a 14-fold increased expression of *CFH* when compared to control cells. The mRNA expression of *CFI* was increased more than 100-fold in post-confluent ARPE cells (Figure 10(B)) cultured for an additional 3 (0.44 ± 0.1-fold change) or 5 days (0.52 ± 0.2-fold change), as compared to control cells that did not show any mRNA expression of *CFI*. Post-confluent ARPE cells co-cultured for 5 days (0.24 ± 0.1-fold change) showed an 80-fold higher *CFI* mRNA expression than that of the control cells.

### Detection of fluid-phase C3a and sC5b-9 by ELISA

**Figure 11.**
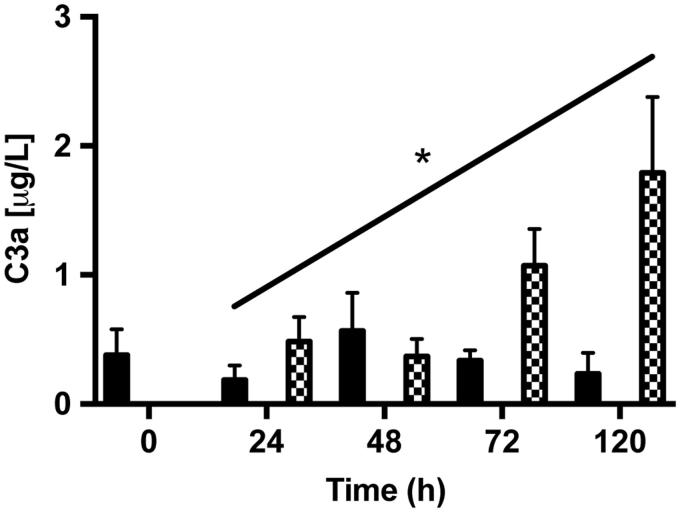
ELISA assays of fluid-phase anaphylotoxin C3a in conditioned medium from post-confluent ARPE cells (black bars), cultured retinas (white bars), and co-cultures (dashed bars). After 5 days in culture, there was a trend toward a slight increase in C3a from the co-cultured specimens over time in culture. C3a could not be detected in the cultured retinas. Data are expressed as means ± SEM; *P < 0.05; *n* = 4.

To detect complement activation products in conditioned medium taken from post-confluent ARPE cells and from cultured as well as from co-cultured retinas, we measured the amount of secreted complement anaphylotoxin C3a and sC5b-9 by using sandwich ELISA. The anaphylotoxin C3a could be detected, although in very low amounts, in medium collected from post-confluent ARPE cells and from co-cultures (Figure 11). The amount of C3a showed a tendency to increase over time in the co-cultured samples after 5 days, with the amount of C3a increasing almost 4-fold when compared with the amount secreted after 1 day (1.80 ± 0.9 μg/L versus 0.48 ± 0.2 μg/L). The terminal complement complex sC5b-9 was not secreted in any measurable amount by any of the cell samples (data not shown).

## Discussion

The complement components present in the retina and retinal surroundings are of essential importance for retinal neurodegeneration. The experimental co-culturing system used in our experiments, i.e. culturing adult porcine retinas with post-confluent ARPE cells, offers a controlled *in vitro* system that can mimic a diverse set of retinal diseases. By using the adult porcine retina we come closer to its human counterpart, as the porcine and human retinas share similarities that include size, architecture, and holangiotic blood supply (76,77). In addition, the porcine retina is trichromatic with a cone-enriched visual streak (67,68,76,78), like the human macula. The adult co-culture system presented here is beneficial for investigating neurodegenerative diseases, in that it allows for investigation in a controlled environment in which post-mitotic cells age with time, synaptic contacts are lost, and apoptosis is an inevitable consequence of age (78). In our experiments the adult porcine retinal photoreceptors were allowed to interact with the apical part of old ARPE cells, mimicking retinal degenerative diseases. Hence, culturing the retinal tissue over time *in vitro* led to a neurodegenerative process equivalent to a retinal degenerative process *in vivo* (69,79,80). In addition, co-culturing the adult retina with ARPE cells decreased the amount of cell death, indicating that ARPE cells are beneficial for culture. These findings are well-known features that are seen when retinas are co-cultured with stem cells and progenitor cells (69,79). However, the degenerative processes are more predominant than the beneficial ones in cultures more than 3 days old.

Chronic complement activation is a known feature seen in different eye diseases. In a pathological state, the quantities of active complement system components increase systemically as well as locally in both the retina and the surrounding tissue (6). Thus, the tissues may be exposed to an overactive complement system that can be detrimental for both the retinal cells and the surrounding tissue. The culture system described here allowed us to assess this process by measuring complement proteins.

In the current study we demonstrate, for the first time, that activated resident microglial cells express C1q, and C1q may possibly be secreted by these cells, since a significantly increased amount of C1q was measured after 5 days in cell culture medium collected from the cultured control retinas. Increased expression of C1q has been well documented in various neurodegenerative diseases (6,81) and shown to co-localize with pathological attributes. Glaucomatous animal models have shown increased labeling of C1q in ischemic ganglion cells and the nerve fiber layer (28), and C1q has been connected to microglial cells and suggested to be a primary microglial activator following retinal ischemia/reperfusion injury (73). To our knowledge, C1q has only been confirmed in drusen of AMD patients (51,82). Also, prior research performed in animal models have been unable to differentiate between resident and migratory microglial cells (73). Blood vessel-derived endothelial cells express C1q (83). C1q has been shown to be involved in angiogenesis (84), and blood vessels in non-cultured retinal tissue were herein C1q-positive. The experiments conducted here support the contention that C1q is expressed in active, resident microglial cells, since the blood-supply could be eliminated as a source during our experimental procedure.

Resident microglial cells use their long protrusions to screen the retinal environment, and actively migrating and phagocytic microglia retain an amoeboid morphology, possibly due to retinal cell degeneration and death (72). This known feature of active microglia in retinal degenerative disorders shows that the experimental model we have developed may mimic those characteristics and be used for further investigation of them.

Recent research (85) has shown that AMD patients have C3 immunoreactive microglia/macrophages in the inner retina. C3 has also been found in drusen as well as in AMD lesions (51). Current findings herein show minor C3 immunoreactivity in ganglion cells after 5 days in culture as well as a C3-positive outer retinal immunoreactivity. The outer C3-reactivity may indicate that C3 is deposited from the ARPE onto the retina during culture. This assumption is further supported by the fact that no secreted C3 could be measured in the conditioned media from the retinal cultures, but only in the media from the co-cultured specimens and from the ARPE cells cultured alone. Antibodies used in these experiments show reactivity against human as well as against swine C3 (86).

Our findings that C4-reactivity is concentrated in the basal part of the ARPE cells may be due to the experimental conditions. Prior research has shown mRNA transcripts of C4 in RPE and choroid (51), and recent research has shown an upregulation of C4 mRNA in RPE/choroid in light-induced photoreceptor damage (87), although this possibility requires further investigation. Our data support the conclusion that there is a local production of CS proteins in the retinal tissue and the ARPE cells. In addition, the culture system used in our experiments did not contain any source of complement or any complement-producing factors other than cells and tissue themselves.

Given that CS homeostasis and protection of self-tissue need to be tightly regulated by endogenous regulators, we investigated soluble regulators (C4BP, CFH, and CFI) in condition media from cultures over time, assessed by Luminex. The classical pathway regulator C4BP and the regulators of the alternative pathway CFH and the protease CFI were secreted from ARPE cells alone, as well as from the co-cultures. None of the regulators was secreted in any notable amount from the cultured control retinas. The absence of regulators in the culturing system controls (retinal explants alone) may reinforce the lack of need for protection in an *in vitro system* and/or suggest that none of the retinal cells need, or are able, to produce these regulators. Also, it may indicate that the absence of RPE cells *in vivo* makes the retinal tissue and surroundings more susceptible to complement attack.

RPE cell degeneration and death are relatively early findings in retinal degenerative diseases as AMD (43,46), and dying cells are known to downregulate complement regulators (29). Polymorphism in the genes encoding complement regulators, such as *CFH* and *CFI*, has been shown to be connected to AMD (6). Other reports have shown connections between AMD and abnormal complement regulatory functions at the cell surface (88–90). Together, these factors may contribute to the presence of decreased amounts of regulators, and consequently increased and inappropriate complement control.

The alternative pathway has been shown to be involved in retinal degenerative diseases and specific regulators CFH and CFI. These findings support the conclusion that complement regulators are affected during the degenerative process at both the gene and protein levels.

Complement anaphylatoxins and terminal complex formation indicate that there is an ongoing activation of the complement system, and these fragments and factors have been connected to a diverse set of degenerative ocular diseases (6,47,51,53,54,91–95). Anaphylatoxins are known chemotactic agents and potent mediators of inflammation (2). The soluble anaphylatoxin C3a was secreted in small amounts from cultured ARPE cells and from the co-cultures. No secretion could be measured from the cultured control retinas, and antibodies used have shown cross-reactivity with swine C3a, investigated by western blot (unpublished data in the group of K.N. Ekdahl). C3a has been shown to reduce proteasome activity in human RPE cells (96,97), which may indirectly contribute to AMD pathology. Reduced proteasome activity is known to enhance the expression of vascular endothelial growth factor (VEGF) (97) and thereby affect angiogenesis, and it is implicated in neovascular AMD. C3a-reduced proteasome activity has also been shown to be connected to light-regulatory pathways in RPE cells and therefore involved in light-induced damage (61,98) and lipofuscin accumulation in RPE cells (99), both of which are factors known to cause oxidative stress in RPE cells and possibly drusen build-up. These regulatory pathways, though potentially seen in light-exposed co-cultures, are not the focus of our study and will need further investigation.

Complement anaphylatoxin receptors transmit the responses provoked by C3a and C5a binding. These anaphylatoxins are potent effectors of inflammation and act on their respective receptors in the nanomolar range (100). C5aR has been implicated in the recruitment of microglial cells in a light-damaged mouse model of AMD and co-localizes with the microglial marker IBA-1 (101). However, in our adult porcine retinas, the C5aR expression was confined to the outer plexiform layer (OPL), Müller glial cells, as well as the inner retinal tissue.

In our hands, the assembly of the terminal complement complex sC5b-9 could not be measured in any sample. Hence, our culture setup did not stimulate the secretion of sC5b-9 during the culture period, although positive sC5b-9 labeling was seen in the blood vessels of the inner control and sparse labeling was seen in ganglion cells after the retinas were cultured for 5 days. The locally generated complement effector molecules probably contributed to the pathological process in the light-exposed post-confluent ARPE cells and the degenerating retina. Thus, although there has been considerable research conducted and knowledge acquired concerning the involvement of diverse complement components in retinal degenerative diseases, much is yet to be learned about how complement operates at the local level. The experimental model described here can be used as a system for complement-based investigations *in vitro*.

## Supplementary Material

Supplemental data
