# Supplementary figures and images for: A model to study complement involvement in experimental retinal degeneration
Source: Ups J Med Sci. 2018 Feb 13;123(1):28–42. doi: 10.1080/03009734.2018.1431744 (PMC5901466; doi:10.1080/03009734.2018.1431744)

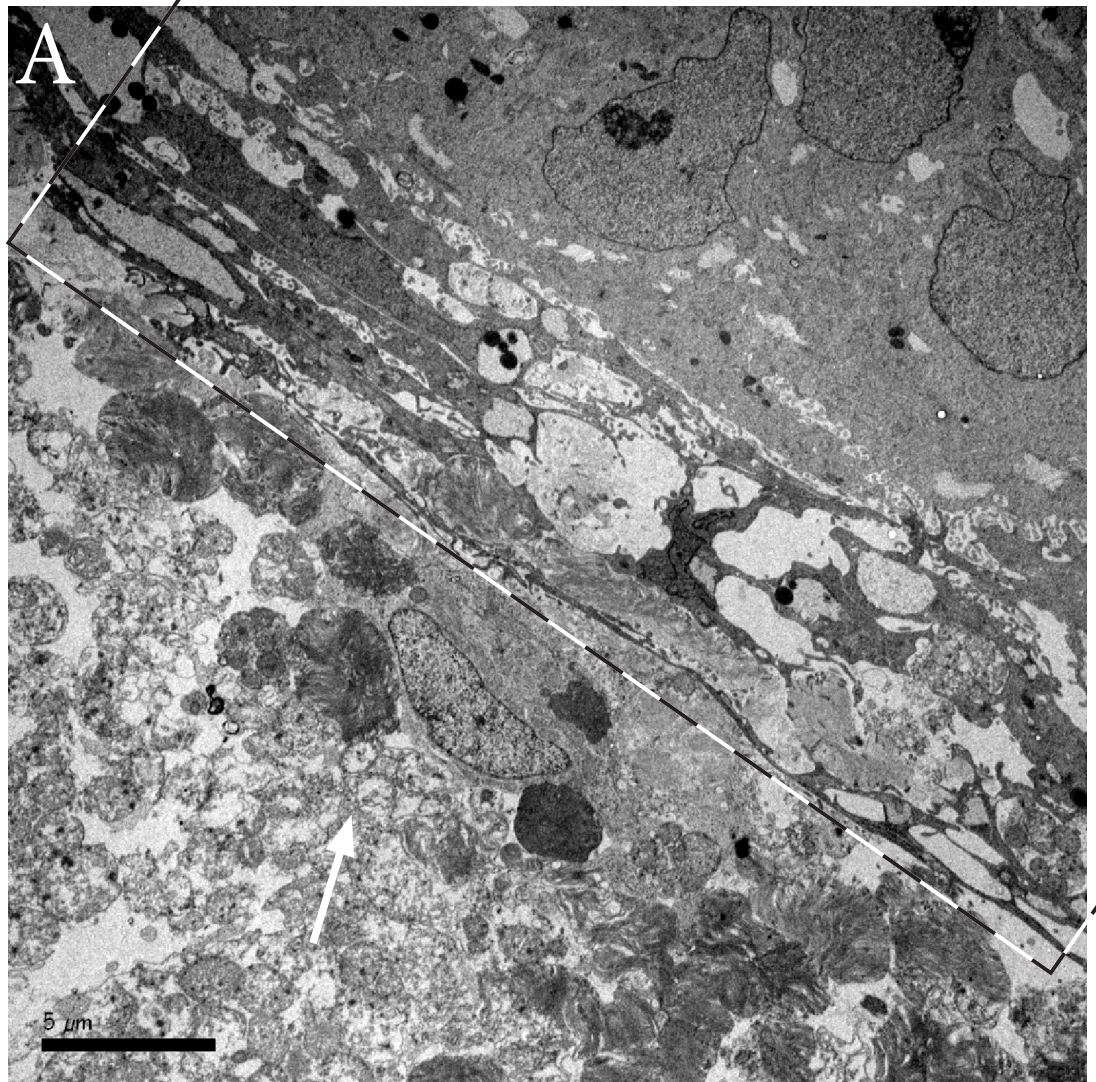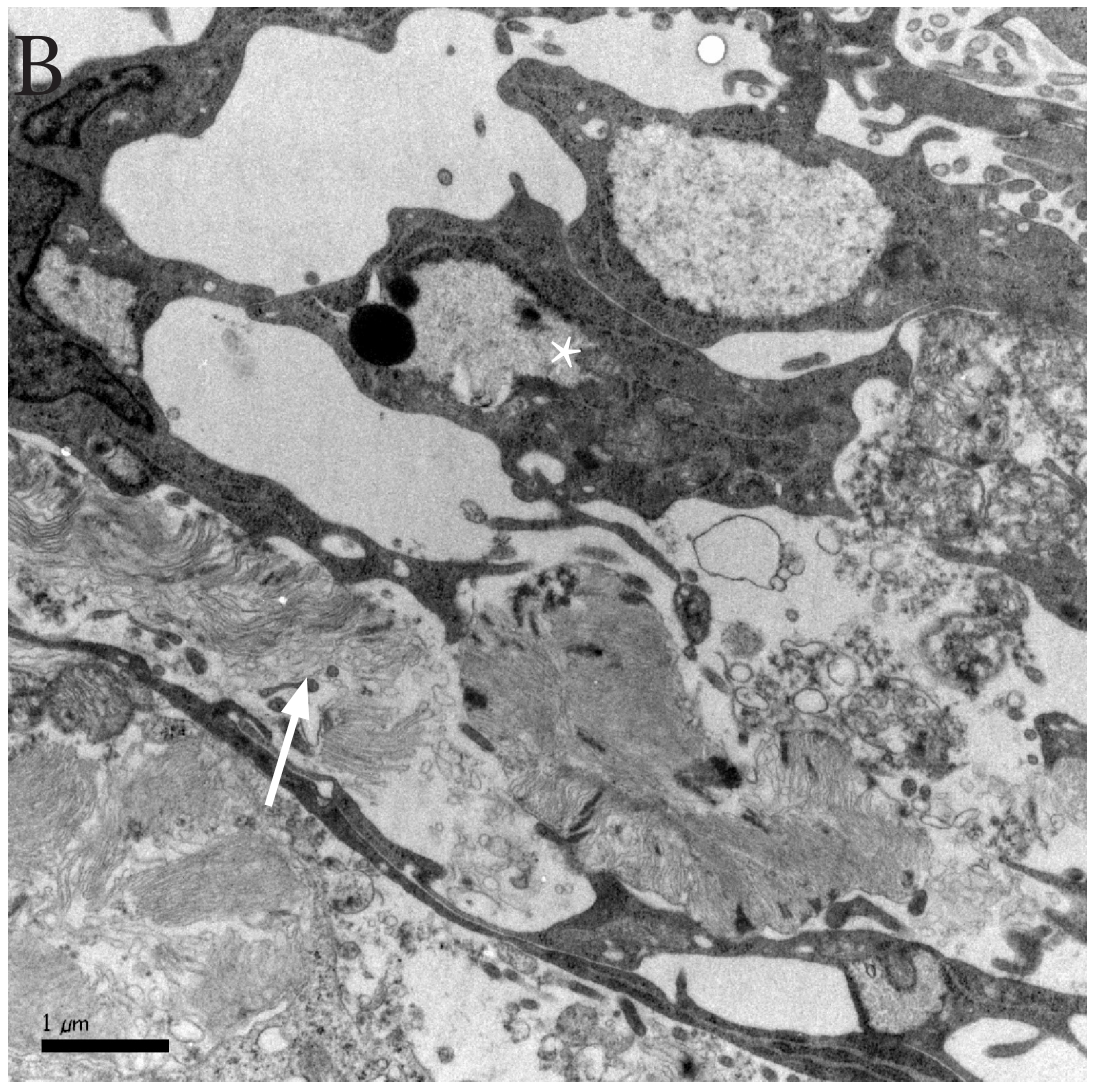

Supplement: Supplemental data [file IUPS_A_1431744_SM4992.pdf]
